# Probiotics and Polycystic Ovary Syndrome: A Perspective for Management in Adolescents with Obesity

**DOI:** 10.3390/nu15143144

**Published:** 2023-07-14

**Authors:** Valeria Calcaterra, Virginia Rossi, Giulia Massini, Francesca Casini, Gianvincenzo Zuccotti, Valentina Fabiano

**Affiliations:** 1 Pediatric and Adolescent Unit, Department of Internal Medicine, University of Pavia, 27100 Pavia, Italy; valeria.calcaterra@unipv.it; 2 Pediatric Department, Buzzi Children’s Hospital, 20154 Milan, Italy; virginia.rossi@unimi.it (V.R.); giulia.massini@unimi.it (G.M.); francesca.casini@unimi.it (F.C.); gianvincenzo.zuccotti@unimi.it (G.Z.); 3 Department of Biomedical and Clinical Science, University of Milano, 20157 Milan, Italy

**Keywords:** polycystic ovary syndrome, obesity, adolescents, dysbiosis, probiotics, microbiome

## Abstract

Polycystic ovary syndrome (PCOS) affects a considerable percentage of females of reproductive age. PCOS is an obesity-related condition and its effects are greatly amplified by obesity. Even though the pathogenesis of PCOS remains complex and has not been fully elucidated, a link between obesity, PCOS, and dysbiosis has been described. The potential role of the gut microbiota in the development and progression of PCOS and its associated symptoms has also been reported. The aim of this narrative review is to present a non-systematic analysis of the available literature on the topic of probiotics and PCOS in adolescents with obesity in order to revise the beneficial effects of probiotics/symbiotic supplementation on hormonal and metabolic profiles and inflammatory conditions. The effectiveness of probiotics/synbiotics in PCOS has been supported. The literature suggests that probiotic/symbiotic supplementation may ameliorate hormonal profiles, inflammatory indicators, and lipid metabolism disturbances caused by PCOS. Studies also show improvements in weight, BMI, insulin, and HOMA-IR, including a potential role it plays in protecting fertility. Even though further studies are needed to confirm these findings, particularly in adolescent patients, probiotic supplementation may be considered a solution for managing PCOS in adolescents with obesity.

## 1. Introduction

Polycystic ovary syndrome (PCOS) affects a considerable percentage of females of reproductive age, with estimates ranging from 3.6% to 15% [1,2]. PCOS prevalence rates vary due to different studies using distinct diagnostic criteria. The National Institute of Health (NIH) criteria of 1990 were the most stringent, defining PCOS as the presence of clinical or biochemical hyperandrogenism and oligoanovulation without other endocrinopathies [1]. However, the Rotterdam Consensus of 2003 broadened the criteria by including the presence of two of the following: clinical or biochemical hyperandrogenism, oligo-anovulation, and polycystic ovary morphology on ultrasonography (PCOM) [1,3]. Subsequently, additional diagnostic criteria emerged, including the Androgen-Excess Society criteria, which require the presence of hyperandrogenism along with oligoanovulation or PCOM. Recently, international evidence-based guidelines have endorsed the utilization of the Rotterdam criteria proposed in 2003 for diagnosing PCOS in women [4]. These changes highlight the importance of standardized approaches for evaluating the prevalence and phenotypes of PCOS.

Obesity is believed to play a crucial role in the development of PCOS, as many women with PCOS are reported to be overweight or obese [5]. Obesity is characterized by an excessive accumulation of fat, resulting in negative health consequences, as it is a risk factor for many diseases, including a broad spectrum of endocrine and reproductive disorders [5]. Globally, it is a growing problem [6,7]. According to the World Health Organization (WHO), in 2016, the global prevalence of overweight and obesity in females aged 5 to 19 years was reported to be 18%, which has markedly increased from the 4% prevalence recorded in 1975 [6,8]. There is still evidence that obesity may contribute to a more severe presentation of PCOS [5].

There are different mechanisms mediating the link between obesity and PCOS, such as the metabolic effects of insulin resistance, steroidogenic and reproductive effects of hyperinsulinemia, adipokine secretion, changes in energy expenditure changes, and physical inactivity [9]. 

Even though the pathogenesis of PCOS remains complex and not fully elucidated, a link between obesity, PCOS, and dysbiosis has been described in human and animal models [10,11,12,13,14], and the role of the gut microbiota in the development and progression of PCOS has been demonstrated [15,16,17].

The aim of this narrative review is to present a non-systematic analysis of the available literature on the topic of probiotics and PCOS in adolescents with obesity in order to revise the beneficial effects of probiotics/symbiotic supplementation on hormonal and metabolic profiles and inflammatory conditions. 

## 2. Methods

**Figure 1:**
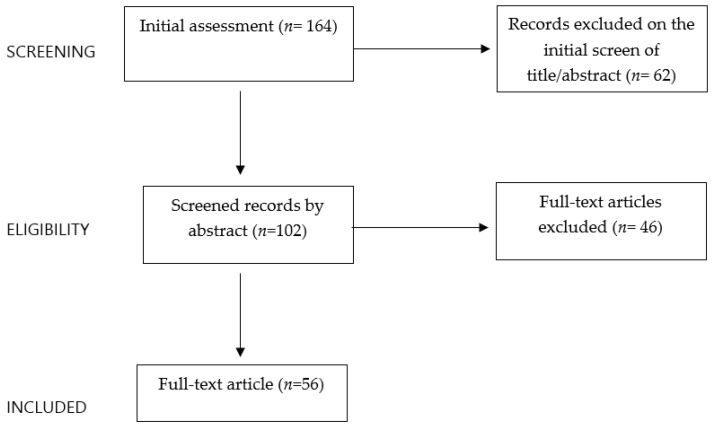
Flowchart of the criteria used for article selection.

A set of inclusion criteria was defined to perform the narrative review as follows: English language articles, meta-analyses, clinical trials, and reviews relevant to this topic in the last twenty years. Case reports and series were excluded. The electronic databases PubMed, Scopus, and Web of Science were used to perform this review. The keywords used for this research alone or/and in combination were polycystic ovary syndrome, adolescent, dysbiosis, microbiome, gut, pediatric, probiotics, obesity, diet, nutrition, obesity lifestyle intervention, and menstrual irregularities. Starting with a total of 164 papers, the authors independently evaluated the abstracts (*n* = 102) and assessed the full texts to identify potentially relevant studies available in the literature (*n* = 56) (Figure 1). The reference lists of all the articles were also checked to identify relevant studies. 

## 3. Polycystic Ovary Syndrome in Adolescents

PCOS is a widespread endocrine-metabolic disorder that affects 5–10% of adult women and 3.4–19.6% of adolescent girls, with variability depending on the diagnostic criteria applicated and the type of population evaluated [18,19,20].

PCOS is a combination of biochemical (elevated androgen), clinical (hyperandrogenism and anovulation), and ultrasound (polycystic ovaries) characteristics [21].

Although the exact pathogenesis and etiology are still unclear, a complex interaction between environmental features, endocrine factors, and genetic predisposition is suspected to be the main etiopathogenetic factor [22]. 

Environmental factors, including socioeconomic level, toxins, stress, physical activity, and food habits, are important determinants in PCOS pathogenesis [23].

Stress can induce psychological stress and overeating disorders [24]. Overeating disorders and obesity may affect oogenesis due to epigenetic modifications of the hypothalamic-pituitary-gonadal (HPG) axis and increased levels of anti-Mullerian hormone (AMH), which inhibits aromatase, leading to hyperandrogenism [25].

Insulin resistance associated or not associated with obesity is considered a fundamental etiological factor, as hyperinsulinemia leads to increased ovarian and adrenal gland secretion of androgens, thereby influencing the development of PCOS [26].

Moreover, the development and symptoms of PCOS usually have a familiar aggregation, suggesting the presence of a genetic predisposition. Daughters of mothers with PCOS have a five-fold increased risk of developing PCOS in their life [27]. 

The genetic predisposition theory considers that polymorphisms in genes involved in steroidogenesis, gonadotrophin release, adipose tissue metabolism, and insulin secretion play a key role in this process [28].

In particular, epigenetic changes in genes encoding CYP11A1, CYP17A1, and CYP19 may influence the risk of developing PCOS [28].

According to the LH-theca interstitial hypothesis, patients with PCOS have elevated serum LH levels, which stimulate theca cells to produce androgens, leading to hyperandrogenism, which is one of the most relevant manifestations of PCOS [29].

As a multifactorial condition, the pathogenesis of PCOS is usually described as a “two-hit” theory. 

Actually, “the first hit” is represented by the genetic predisposition, and “the second hits” is a trigger factor, which is usually acquired [30].

According to the NIH criteria in 1990, the diagnosis of PCOS in adult women was based on biochemical or clinical evidence of hyperandrogenism and ovulatory dysfunction [31]. PCOM was not included in these criteria because of the knowledge that the polycystic aspect of the ovaries is observed in 20–30% of healthy women [32].

The NIH criteria were successively substituted in 2012 using the Rotterdam criteria. Rotterdam criteria defined PCOS as the presence of two out of the three following features: hyperandrogenism, PCOM, and oligoanovulation [33].

Although both the NIH and Rotterdam criteria were based on adult women, these criteria have been applicated to adolescents in clinical practice. 

Adolescence, defined by the WHO as the period between 10 and 19 years of age, is characterized by psychological and physical changes, including menstrual irregularities, PCOM, and hyperandrogenism, making the diagnosis of PCOS controversial in adolescents [34,35,36].

Over the last decade, three international consensuses of PCOS in adolescents recommended the use of NIH diagnostic criteria rather than Rotterdam criteria. Rotterdam criteria, which have PCOM as a diagnostic feature, were considered inappropriate for adolescents due to the poor specificity that may lead to the overdiagnosis of PCOS. A transient polycystic ovarian morphology may be found in many healthy girls, especially in the first years after menarche [37]. Abdominal ultrasound, which is most frequently used in adolescence, has less accuracy in evaluating ovarian morphology than transvaginal ultrasound [38]. 

These three recommendations include only ovulatory dysfunction and clinical or biochemical hyperandrogenism as the diagnostic criteria for adolescent PCOS, excluding other conditions that are similar to PCOS [38,39,40] (Table 1).

Ovulatory dysfunction is defined as abnormal menstrual cycles in relation to the time after menarche, but menstrual irregularities are described differently, although all the authors agree that measles for >90 days one year after menarche must be considered a risk factor for PCOS and require further investigations.

Moreover, ovulatory dysfunction also includes amenorrhea, which can be described as the absence of menarche at 15 years of age or 2–3 years after thelarche or the absence of menarche in girls who completed puberty.

Regarding hyperandrogenism, all the authors define it clinically as hirsutism, which can also be mild [38], in contrast to previous recommendations [26].

All guidelines agree that elevated testosterone-free or total testosterone levels can be considered a marker of hyperandrogenism [38,39,40].

Moreover, PCOM, insulin resistance, and hyperinsulinemia alone are not considered diagnostic criteria for PCOS, although they are common clinical manifestations in girls with PCOS [40].

The management of PCOS in adolescents is challenging. The purpose of treatment is to improve the hormonal status and quality of life of adolescents and prevent complications.

The first-line treatment is represented by dietary and lifestyle changes and weight loss, and 40–70% of adolescents with PCOS are overweight or obese [41].

These treatments have been demonstrated to reduce androgen levels and menstrual irregularities, increase metabolism in overweight girls, and decrease the risk of developing metabolic syndrome in girls with normal weight [40,42].

Regarding pharmacological treatments, metformin, estrogen-progestin contraceptive pills, and anti-androgens are the main options [38].

COCPs contain both ethinylestradiol and progestin, but progestin with antiandrogenic activity may also be used.

The aim of COCP is to regulate hyperandrogenism and improve menstrual irregularities; therefore, they are considered the first-line treatment option, although they are associated with side effects that must be considered before starting therapy.

Progestin pills with antiandrogenic activity, such as cyproterone acetate, spironolactone, and flutamide, are associated with fewer side effects; however, due to the lack of estrogens, the decrease in free androgen index is not as effective as COCP [40].

Due to their teratogenic effect, it is recommended to associate antiandrogens with COCP [40,43].

Metformin can be considered in adolescents with obesity and insulin resistance if lifestyle changes are ineffective.

Usually, metformin is used in adolescents with a BMI higher than 25 added to COCP. Metformin induces ovulation and reduces serum androgen levels and insulin resistance [22,40]. The association of metformin with insulin-sensitizing drugs, such as thiazolidinediones, has also been proposed, but its use in adolescents is controversial.

Moreover, new therapeutic options have been developed. Trent et al. reported that the use of N-acetylcysteine induces a decrease in hyperinsulinemia and testosterone levels with high tolerability [43].

Also, myoinositol and vitamin D have been shown to be useful for inducing menstrual cycle regulation and weight reduction [44,45].

Other studies have reported beneficial effects of carnitines (N-acetyl-carnitine and L-carnitine) in women with PCOS and hyperinsulinemia. Carnitines are involved in the fatty chain transport into the mitochondria, stimulating fatty chain oxidation and removing products of metabolism from the cytoplasm [46].

The optimal pharmacological treatment for young women with PCOS is still controversial due to the lack of studies in the literature, suggesting that further trials are needed to provide a reliable and safe treatment for adolescents with PCOS.

## 4. Polycystic Ovary Syndrome and Obesity

As early as 1935, Stein and Leventhal [47] first described PCOS as a reproductive disorder characterized by enlarged smooth polycystic ovaries, menstrual irregularity (amenorrhea or occasionally menometrorrhagia), infertility, and hirsutism [47,48]. In the 1950s, hormonal features of PCOS, characterized by altered gonadotropin secretion and androgen production, were defined [48]. Since the 1950s, researchers began to define the closely interrelated reproductive hormonal alterations in PCOS, which, in 1976, Rebar et al. [49] defined as a “vicious cycle” [48,49].

PCOS is characterized by a complex set of hormonal imbalances, which is self-sustaining. Specifically, the LH: FSH ratio increases, and LH release increases, along with reduced FSH values compared to normal menstrual cycles. However, this alteration may not be detectable in a single blood sample because of the pulsatile release of LH [50]. Furthermore, the frequency and amplitude of pulsatile GnRH secretion are increased in PCOS patients. This leads to the selective elevation of LH and suppression of FSH release. Free testosterone levels are elevated due to reduced levels of sex hormone-binding globulin (SHBG) [48,51,52]. Estradiol levels are consistently mid-follicular, and progesterone levels are low in anovulatory PCOS [48]. Specifically, LH stimulates testosterone production by ovarian theca cells, whereas reduced FSH results in decreased aromatization of testosterone to estradiol by granulosa cells. In the ovary, theca cells also exhibit a constitutive increase in steroidogenic enzyme pathways involved in androgen biosynthesis. These enzymes are shared with the adrenal gland and potentially contribute to the increased adrenal androgen production observed in PCOS. Anti-Müllerian hormone (AMH), produced by granulosa cells, plays a role in folliculogenesis, and its increased levels in PCOS reflect disruption of folliculogenesis [53,54]. Moreover, PCOs are characterized by increased antral follicles, ovarian stroma, theca cell hyperplasia, and ovarian cortical thickening [48]. Theca cells in PCOS produce higher levels of androgens, both at baseline and in response to LH, indicating the constitutive activation of steroidogenic enzymes [48,55]. This increased androgen production is observed in women with PCOS, both with and without ovulation [55]. Furthermore, since its original description by Stein-Leventhal in 1935, a common feature of PCOS is obesity, particularly visceral obesity, which amplifies and worsens all the metabolic and reproductive outcomes of PCOS [56].

In fact, several studies have observed a strong correlation between PCOS and obesity; more specifically, in the United States, it has been observed that up to 80% of women with PCOS are overweight or obese [5,57]. Similar patterns have been reported in other countries, with rates ranging from 30% to 50% among women with PCOS [5]. Moreover, even if there is a lack of community-based studies on the prevalence of PCOS in adolescent girls, available reports indicate that a significant proportion of adolescent PCOS patients, ranging from 30% to 40%, are overweight or obese [1,58,59]. Some authors argue that the high proportion of overweight or obese women with PCOS may be overestimated due to a referral bias [60].

Adipose tissue functions as an active endocrine organ, producing and releasing biologically active molecules known as “adipokines”, which are a subgroup of cytokines specifically produced and secreted by the adipose tissue [61,62]. Specifically, adipokines are involved in both pro-inflammatory activities (leptin, resistin, osteopontin, interleukin (IL)-6 and -10, tumor necrosis factor (TNF-α, etc.), and some anti-inflammatory actions (such as adiponectin and omentin) [63]. These adipokines play crucial roles in regulating various physiological processes, including energy metabolism, appetite regulation, insulin sensitivity, inflammation, atherosclerosis, and reproduction [64]. In particular, adiponectin, which is an adipokine predominantly synthesized and released by white adipose tissue, exhibits higher production and secretion in visceral fat compared to subcutaneous fat [64,65]. It has anti-inflammatory, anti-atherogenic, and insulin-sensitizing properties and plays a role in modulating reproductive functions [64].

Obesity leads to structural and functional changes in adipose tissue, which is characterized by hypertrophic adipocytes, resulting in a state of hyperinsulinemia, hyperlipidemia, hyperleptinemia, and chronic low-grade inflammation [66]. This chronic low-grade inflammatory state is characterized by immune cell infiltration, particularly by macrophages, in adipose tissue [67]. Activated macrophages secrete pro-inflammatory cytokines (TNF-α, IL-6, and IL-1) [66]. Activated macrophages also infiltrate other organs and may contribute to the development of insulin resistance. The density of adipose tissue macrophages is similar between individuals with PCOS and controls [5,68].

Another notable aspect of obesity is IR, which leads to an accelerated breakdown of adipose tissue, particularly in visceral adipocytes [69,70]. Women with PCOS also exhibit a significant increase in adipocyte lipolysis induced by catecholamines, and there is a correlation between adipocyte size and lipolytic responsiveness [5,71].

It is crucial to highlight that females with PCOS present with marked IR, as shown in studies on glucose metabolism [48,58], and it appears to be pivotal in PCOS pathogenesis [1]. IR and hyperinsulinemia can be observed in adolescent girls with PCOS, although it is important to consider that puberty itself is associated with physiological IR [5]. Furthermore, obesity has an additional impact on IR in adolescents with PCOS, exacerbating both metabolic and reproductive abnormalities [72]. IR is frequently observed in patients with PCOS, with a prevalence ranging from 44% to 70%, and is more pronounced in individuals with PCOS and obesity [69]. Specifically, research has shown that women with PCOS and IR generally have a higher BMI, greater visceral fat distribution, elevated androgen levels, and more severe symptoms of PCOS, including hirsutism, acne, and ovulatory dysfunction, than women with PCOS without IR. The role of insulin extends beyond metabolic processes to ovarian physiology and pathophysiology [5,69]. Insulin receptors are present in ovarian cells, where they influence follicular development and steroidogenesis. Consequently, hyperinsulinemia leads to increased androgen production, exacerbating insulin resistance in a self-perpetuating cycle. In addition, insulin suppresses the production of SHBG, resulting in increased levels of circulating free testosterone and contributing to hyperandrogenism [73]. Insulin also affects adrenal androgen production, with some women exhibiting excessive adrenal steroidogenesis [74,75]. Although insulin resistance is partly independent of obesity, the presence of obesity amplifies insulin resistance and hyperandrogenism in PCOS [5]. Nevertheless, it is noteworthy that, although the current diagnostic criteria for PCOS do not explicitly include obesity or IR, their coexistence increases the risk of metabolic complications such as metabolic syndrome, type 2 diabetes mellitus, and dyslipidemia in individuals with PCOS [1,58,59].

**Figure 2:**
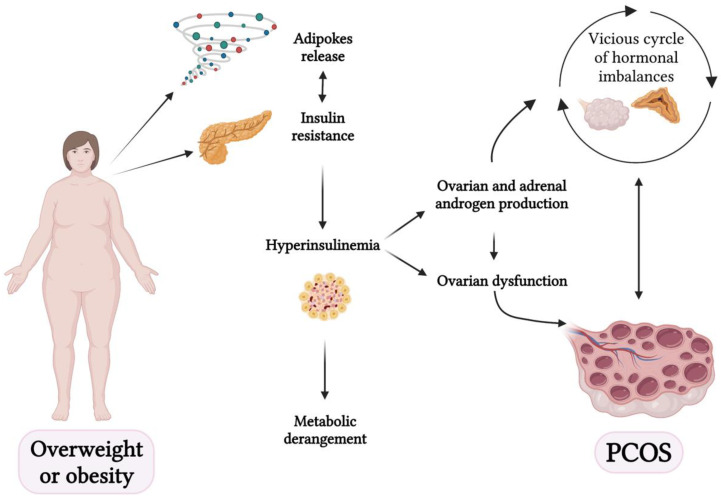
The link between microbiome and polycystic ovary syndrome (PCOS) (created with BioRender.com (accessed on 2 April 2023)).

In Figure 2, the link between obesity and PCOS is shown.

## 5. Dysbiosis and Polycystic Ovary Syndrome

The g microbiome includes diverse bacteria, archaea, viruses, fungi, protozoa, and their metabolites, which have gradually adapted to live on the mucosal surface of the intestine or in its lumen [76,77]. The microbiota is acquired primarily at birth and then stabilizes at around 3 years of age [77,78]. Although the taxonomic composition of the gut microbiota is highly variable, even among healthy individuals, its function is similar in all individuals [79]. It plays a key role in host physiology, immune regulation, gastrointestinal epithelial barrier function, endocrine system, host metabolism, and production of vitamin B12 and short-chain fatty acids (SCFAs) via fermentation and neurological functions [77,80,81]. The four most prevalent bacterial phyla are *Bacteroidetes* and *Firmicutes*, *Actinobacteria,* and *Proteobacteria* [82].

The gut microbiota tends to be stable, but it can be affected by several factors, such as lifestyle, exposure to chemicals in the environment, age, antibiotic use, stress, and changes in diet [77,78,83].

Changes in the gut microbiome, described as dysbiosis, have been associated with autoimmune diseases, neurological and cardiovascular disorders, and metabolic impairments, including PCOS [15].

In 2012, a link between dysbiosis and the metabolic and reproductive features of PCOS was proposed for the first time [16]. Kelley et al. and Lindheim et al., in 2016 and 2017, respectively, described that changes in the gut microbiome are associated with PCOS in both mice models and women [17]. Then, many other studies on humans and animals reported further evidence of the association between gut dysbiosis and PCOS [10,11,12,13,14].

Several studies have compared the microbiota of PCOS patients with that of control groups, indicating that changes in the composition of the gastrointestinal microbiome and gut dysbiosis may play a significant role in the development of PCOS. Specifically, a consistent finding across these studies is the reduced α diversity observed in the PCOS group compared to control populations [13,77,84,85,86,87]. Moreover, significant compositional changes have been reported between the PCOS and control groups before and after probiotic administration [88]. While reduced α diversity is commonly observed in women with PCOS, no specific bacterium has been identified as a causal factor [10,13,77,85,89]. Some studies suggest an expansion of species associated with mucosal inflammation and the production of pro-inflammatory cytokines and chemokines, such as *Prevotella* and *Escherichia coli*, as well as other Gram-negative bacteria that produce lipopolysaccharides (LPS) [77,84,89]. However, different compositional changes have been identified in other studies [85,90]. These discrepancies may be attributed to the lack of standardization in host variables, including diet, geographic variations, obesity rates in case and control subjects, functional redundancy, or differences in microbiota assessment techniques [77,91].

Two principal hypotheses were proposed regarding the role of dysbiosis in the pathogenesis of PCOS.

Tremellen and Pearce et al. propose that a high-fat and carbohydrate diet may alter the gut epithelium barrier leading to a disruption of the intestinal barrier and a passage of toxins and antigens to the bloodstream inducing a hyperactivation of the immune system [16]. In their recent review, Zhao et al. [92] examined the role of the gut microbiota and tried to identify several other potential mechanisms [77,92]. These mechanisms include enhanced energy absorption, potential effects on short-chain fatty acid metabolism, changes in bile acid metabolism affecting glucose and lipid metabolism as well as inflammation, various physiological effects on choline metabolism pathways, and modulation of gastrointestinal hormones involved in the gut–brain interaction [92]. Understanding these diverse microbial metabolic pathways implicated in PCOS pathogenesis could potentially lead to more targeted treatments, such as utilizing prebiotics, probiotics, fecal microbiota transplantation, and traditional Chinese medicine [92]. Furthermore, Rizk et al. [91] investigated a series of metabolites associated with gastrointestinal dysbiosis, including host-derived metabolites (lactate, trimethylamine N-oxide, and primary bile acids), microbiota-associated metabolites (short-chain fatty acids and secondary bile acids), and targeted metabolomics studies. Although our current knowledge is still limited, preliminary findings support the role of dysbiosis in the pathogenesis of PCOS [91]. There may be multiple possible mechanistic pathways, depending on the specific genetic, dietary, environmental, and microbiota characteristics of each individual [91].

This first hypothesis places a particular emphasis on diet and obesity as a fundamental etiopathogenetic factor of PCOS, although not all women with PCOS are obese and the incidence of PCOS is relatively similar worldwide despite differences in diet [93,94]. Indeed, according to the dysbiosis theory, the utilization of prebiotics, probiotics, and synbiotics as treatment options holds promise in restoring eubiosis, reversing pathophysiological alterations, and enhancing the biochemical and clinical features of PCOS [77].

A second hypothesis proposed to explain the role of dysbiosis in the pathogenesis of PCOS suggested that hyperandrogenism can induce an alteration of the gut microbiome due to a direct effect of testosterone as a substrate of gut enzyme and an indirect effect via androgen receptors and immune modulation, independent of diet and obesity [95].

Recent studies have shed light on the close relationship between gut microbiota and normal sex hormone levels in rodent models [96]. To further investigate the intricate interplay between androgens, the microbiome, and PCOS, Han et al. [97] conducted a comprehensive study in rats. In this study, pseudo-germ-free rats were generated via targeted antibiotic treatment to eliminate the existing gut microbiota [97]. These pseudo-germ-free rats were then treated with DHEA and underwent fecal microbiota transplantation (FMT). Notably, in addition to inducing dysbiosis via FMT, researchers observed the development of both endocrinological and metabolic PCOS phenotypes in these rats. These phenotypes were characterized by disruptions in glucose and lipid metabolism, perturbed estrous cycles, the presence of polycystic ovaries, and alterations in reproductive hormones [97]. These findings strongly suggest that gut dysbiosis may collaborate with hyperandrogenism to perturb metabolic and endocrine homeostasis via disturbances in the hypothalamic–pituitary–adrenal (HPA) and hypothalamic–pituitary–ovarian (HPO) axes [97]. However, the precise mechanism by which DHEA directly contributes to the metabolic and endocrine phenotypes of PCOS, and the extent to which this process relies on the presence and modification of the gut microbiota still requires further investigation.

In 2020, Jobira et al. conducted a prospective case-control study on fifty-eight obese adolescent girls to compare the gut microbiome of girls without PCOS with similar activity levels, food habits, and BMI [86]. They first demonstrated that girls with PCOS have a different gut microbiome independent of dietary habits, weight, and physical exercise. Moreover, they supported the hypothesis of an interaction between free serum levels of testosterone and altered gut microbiota, especially a decreased α diversity and a significant alteration of β-diversity [86].

**Figure 3:**
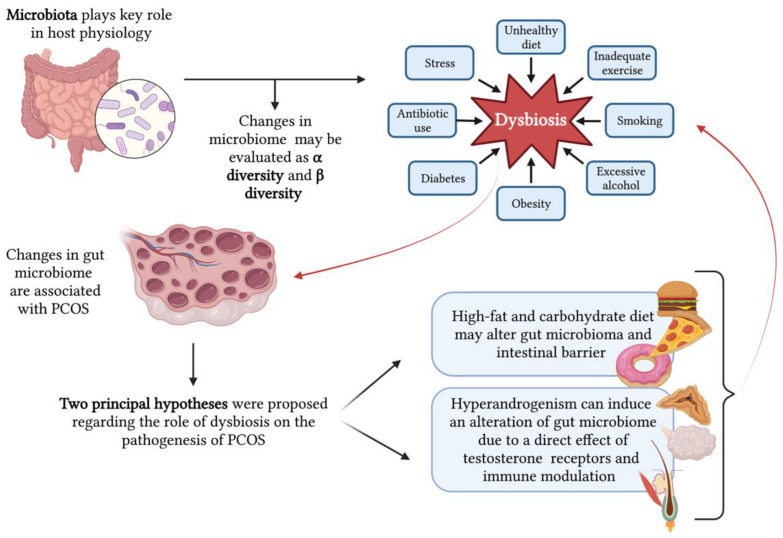
Relationship between microbiome and polycystic ovary syndrome (PCOS) (created with BioRender.com (accessed on 2 April 2023)).

In Figure 3, the relationship between the microbiome and PCOS is shown.

## 6. Probiotics and Polycystic Ovary Syndrome

In recent years, several studies have been conducted to explore the relationship between PCOS and changes in the gut microbiota community. These investigations aimed to better understand the potential role of the gut microbiota in the development and progression of PCOS and its associated symptoms [98].

Studies have indicated that the gut microbiome of women diagnosed with PCOS is characterized by lower diversity compared to women without PCOS. This decrease in α and β microbial diversity has been associated with hyperandrogenism and an elevation in systemic inflammation levels. The decrease in microbial diversity observed in PCOS is often marked by a reduction in beneficial bacteria such as *Lactobacilli* and *Bifidobacteria*. Conversely, there is often an increase in pathogenic bacteria such as *Escherichia* and *Shigella*. There is also an observed alteration in the balance of certain bacterial species, specifically *Bacteroidetes* and *Firmicutes*, leading to altered production of short-chain fatty acids and a negative impact on metabolism, gut barrier integrity, and immunity [99,100].

Gut dysbiosis is considered to be the underlying cause of inflammation and changes in gut mucosa permeability, which can have profound effects on an individual’s overall health and could increase the predisposition to develop pathologies. Evidence suggests that women with PCOS have higher levels of intestinal permeability. It is important to note that the gut microbiota plays a vital role in metabolizing dietary substrates that enter the gut and produce various metabolites that can have direct effects on the intestines or enter the systemic circulation to influence different host tissues, including the ovary, liver, muscle, and adipose tissue, which undergo alterations in their functions in the context of PCOS. Among the gut bacterial metabolites that are known to be altered in PCOS, there are bile acids, short-chain fatty acids (SCFAs), and trimethylamine (TMA). These metabolites have been implicated in the pathophysiology of PCOS and its associated metabolic dysregulation. In addition, gut mucosa permeability alteration allows the passage of lipopolysaccharides (LPS) from Gram-negative bacteria in the colon into the bloodstream. The activation of the immune system triggered by LPS can disrupt the function of insulin receptors, leading to elevated serum insulin levels. Additionally, this immune system activity can promote the production of androgens in the ovaries and interfere with the normal formation of follicles, contributing to the pathogenesis of PCOS [91,99].

Significant efforts have been made to develop innovative strategies for managing PCOS, and probiotics have emerged as a promising tool for its treatment. Probiotic microorganisms are naturally present in fermented foods and possess various beneficial properties [101]. They exhibit antioxidant, antimicrobial, and anti-inflammatory properties, along with the ability to improve metabolic parameters, modulate the composition of the intestinal microbiota, and regulate the immune system. Among the most commonly utilized bacterial genera as probiotics are *Lactobacillus*, *Bacillus*, *Bifidobacterium*, *Streptococcus*, and *Enterococcus*. These probiotic strains have been extensively studied and have shown potential for promoting gut health and overall well-being [102].

Probiotic supplements have been demonstrated to improve the metabolic profiles of patients affected by PCOS. Indeed, in 2022, Tabrizi et al. showed that probiotic supplementation has a significant impact on the regulation of hormonal and inflammatory indicators, with a significant decrease in the free androgen index and malondialdehyde, an increase in SHBG and nitric oxide, and an improvement in the weight, BMI, insulin, HOMA-IR, hirsutism, and total testosterone of PCOS patients. Other recent meta-analyses have confirmed these findings [101,103].

In line with the results of this recent study, many other studies have supported these findings. In 2022, Kaur et al. conducted a randomized, double-blind placebo-controlled study to evaluate the efficacy of multi-strain probiotics (*Lactobacillus acidophilus* UBLA-34, *L. rhamnosus* UBLR-58, *L. reuteri* UBLRu-87, *L. plantarum* UBLP-40, *L. casei* UBLC-42, *L. fermentum* UBLF-31, *Bifidobacterium bifidum* UBBB-55, and fructo-oligosaccharides (100 mg)), dietary and lifestyle modifications on the restoration of menstrual regularity, weight reduction, and metabolic and hormonal profiles in women with PCOS. The study showed that supplementation improved total testosterone levels, waist circumference, waist-to-hip ratio, and menstrual domain of quality of life compared to placebo. No adverse events related to the study were reported [104]. The 2021 study conducted by Chudzicka-Strugała et al. aimed to investigate whether the incorporation of probiotic/synbiotic supplementation alongside lifestyle modifications would result in a more significant decrease in weight and testosterone levels among overweight and obese women with PCOS. It was a randomized double-blind placebo-controlled trial (*n* = 39). Both study groups (placebo vs. intervention) underwent identical lifestyle modifications, which involved closely monitored dietary adjustments and an exercise regimen. The dietary plan involved restricting caloric intake from 1400 to 1800 kcal/day, based on body composition analysis, and providing personalized guidance on food selection and the exclusion of alcohol. The exercise regimen included daily walking sessions lasting 30 to 40 minutes. Participants in the placebo group were administered four placebo capsules daily. On the other hand, participants in the synbiotic group received a synbiotic supplement consisting of four capsules per day. The synbiotic supplement contained the following probiotics: two strains of *Bifidobacterium lactis, Lactobacillus acidophilus, Lactobacillus paracasei*, *Lactobacillus plantarum, Lactobacillus salivarius,* and *Lactobacillus lactis*. Additionally, the symbiotic supplement contained prebiotics, specifically fructo-oligosaccharides and inulin. In the placebo group, a 5% reduction in BMI was associated with significant decreases in waist, hip, and thigh circumferences. On the other hand, the synbiotic group experienced an 8% decrease in BMI, which was significantly greater than that of the control group, and was accompanied by reductions in waist, hip, and thigh circumferences. Testosterone levels did not show a significant decrease in the placebo group (6%), whereas, in the synbiotic group, a significant reduction (32%) in testosterone levels was noted. The decrease in testosterone levels was significantly greater in the synbiotic group compared to the placebo group [105].

Unlike previous studies, Heshmati et al. conducted a meta-analysis of seven RCTs and observed that probiotic supplementation did not have a significant impact on anthropometric measurements, such as weight, body mass index (BMI), and waist circumference, in patients with PCOS compared to placebo. However, they found a significant effect on glycemic control with lower insulin levels and on lipid metabolism, characterized by reduced serum triglyceride (TG) levels and increased high-density lipoprotein (HDL) levels. These findings suggest that probiotic supplementation could be used as an adjunct therapy for managing PCOS [106]. The effects of probiotic therapy on the hormonal profiles of women with PCOS have not yet been widely established [107].

Kwok et al. investigated the effects of the probiotic intervention on inflammation markers in women with PCOS. Researchers administered *L. acidophilus*, *L. plantarum*, *L. fermentum*, and *L. gasseri* at 2 × 10^9^ CFU of each strain per day for 12 weeks in 60 patients. IL-10 showed a significant increase in the probiotics group compared to the placebo group, and IL-6 showed a significant decrease in both groups, without a difference in serum TNF-α. These findings suggest that these four lactobacilli probiotic strains can be useful in modulating inflammation and metabolic dysfunctions [108,109]. 

A systematic review and meta-analysis were conducted with the aim to assess the effects of probiotics, prebiotics, and synbiotics on hormonal and inflammatory indices. The study included a total of 13 studies with 855 participants diagnosed with PCOS, with 438 women in the intervention group and 417 women in the control group. No significant differences were observed between the intervention and control groups in terms of testosterone, dehydroepiandrosterone sulfate (DHEAS), total glutathione, hsCRP, and hirsutism. However, the authors showed that sex hormone-binding globulin (SHBG) and nitric oxide (NO) concentrations increased significantly in the probiotics and synbiotics groups compared to the placebo group. FAI and MDA concentrations in the probiotics and synbiotics groups reduced significantly compared to those in the placebo group [103].

Miao et al. performed a meta-analysis to evaluate the effect of probiotics and symbiotics on insulin resistance in patients with PCOS. The authors selected seven studies comprising 486 patients. The results showed that the intervention significantly reduced HOMA-IR and serum insulin levels but did not affect BMI, WC, hip circumference, or fasting blood sugar (FBS). However, the negative results obtained could be potentially attributed to the short duration of the intervention. It is possible that the optimal treatment duration for addressing central obesity may extend beyond 12 weeks [110].

**Figure 4:**
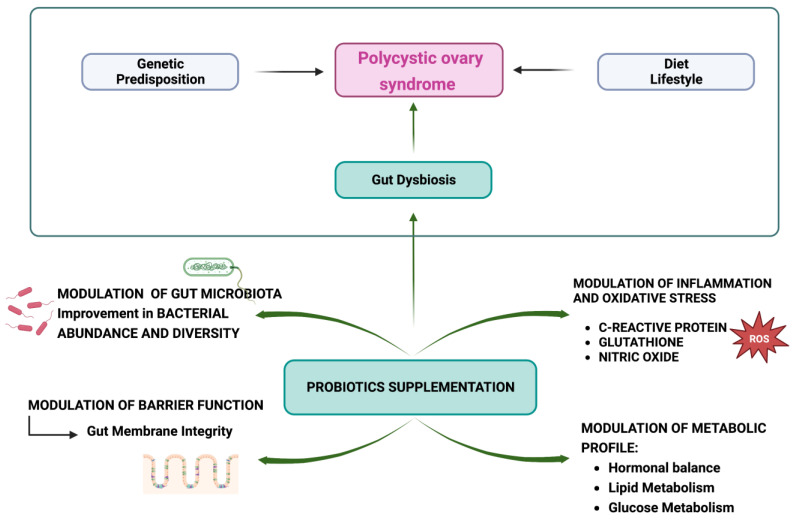
Effects of probiotic supplementation on polycystic ovary syndrome (PCOS) (created with BioRender.com (accessed on 2 April 2023)).

In Figure 4, the effects of probiotic supplementation on PCOS are shown.

## 7. Conclusions

PCOS is an obesity-related condition and its effects are greatly amplified by obesity [74,111]. A link between obesity, PCOS, and dysbiosis, and the evidence of changes in microbiota composition in women with PCOS compared with healthy subjects are reported [16,77,89]. The effectiveness of probiotics/synbiotics in PCOS has been supported. The literature suggests that probiotic/symbiotic supplementation may ameliorate hormonal profiles, inflammatory indicators, and lipid metabolism disturbances associated with PCOS. Studies also show an improvement in weight, BMI, insulin, and HOMA-IR, indicating its potential role to protect fertility. Even though further studies are needed to confirm these data, probiotic supplementation may be considered as a solution for managing PCOS in adolescents with obesity. PCOS may originate in the very early stages of development, showing clinical features later in adolescence; microbiome monitoring and early probiotic supplementation during childhood and adolescence could be useful to modulate dysbiosis in order to prevent it as a modifiable cause of PCOS.

## Figures and Tables

**Table 1:** Consensus for diagnosing polycystic ovary syndrome (PCOS) in adolescents [38,39,40].

Criteria	Pena AS et al., 2020	Ibanez L. et al., 2017	Witchel S. et al., 2015
Menstrual Irregularity	Strong correlation with the timing of menarche: Irregular cycles are normal in the first year after menarche. Menstrual cycles <21 and >45 days 1–3 years post-menarche. Menstrual cycles <21 days and >35 days 3 years post-menarche (<8 cycles per year).	Irregular cycles two years post-menarche.	Cycles <20 days and >45 days two years post-menarche.
Amenorrhea	Primary amenorrhea is defined as amenorrhea at 15 years of age or 3 years post-thelarche.	Primary amenorrhea is defined as amenorrhea in girls who completed puberty.	Primary amenorrhea is defined as amenorrhea in girls who completed puberty.
Clinical hyperandrogenism	Hirsutism is defined as a modified Ferriman Gallway score of 4–6 and/or severe acne.	Hirsutism and/or moderate to severe acne unresponsive to topical therapy.	Moderate to severe hirsutism and acne unresponsive to topical therapy.
Biochemical hyperandrogenism	In females with irregular cycles without hyperandrogenism testosterone, free testosterone of free androgen index can help with diagnosis.	Confirmation test in girls with hyperandrogenism using high-quality assays.	Elevation of total testosterone and/or free testosterone in girls with hyperandrogenism.

## Data Availability

Not applicable.

## References

[1] Dabadghao P. (2019). Polycystic Ovary Syndrome in Adolescents. Best Pract. Res. Clin. Endocrinol. Metab..

[2] Azziz R., Carmina E., Chen Z., Dunaif A., Laven J.S.E., Legro R.S., Lizneva D., Natterson-Horowtiz B., Teede H.J., Yildiz B.O. (2016). Polycystic Ovary Syndrome. Nat. Rev. Dis. Primers.

[3] The Rotterdam ESHRE/ASRM-Sponsored PCOS Consensus Workshop Group (2004). Revised 2003 Consensus on Diagnostic Criteria and Long-Term Health Risks Related to Polycystic Ovary Syndrome. Fertil. Steril..

[4] Teede H.J., Misso M.L., Costello M.F., Dokras A., Laven J., Moran L., Piltonen T., Norman R.J., Andersen M., International PCOS Network (2018). Recommendations from the International Evidence-Based Guideline for the Assessment and Management of Polycystic Ovary Syndrome. Hum. Reprod..

[5] Calcaterra V., Verduci E., Cena H., Magenes V.C., Todisco C.F., Tenuta E., Gregorio C., De Giuseppe R., Bosetti A., Di Profio E. (2021). Polycystic Ovary Syndrome in Insulin-Resistant Adolescents with Obesity: The Role of Nutrition Therapy and Food Supplements as a Strategy to Protect Fertility. Nutrients.

[6] WHO Obesity. https://www.who.int/health-topics/obesity#tab=tab_1.

[7] Sommer A., Twig G. (2018). The Impact of Childhood and Adolescent Obesity on Cardiovascular Risk in Adulthood: A Systematic Review. Curr. Diabetes Rep..

[8] WHO Obesity and Overweight. https://www.who.int/news-room/fact-sheets/detail/obesity-and-overweight.

[9] Barber T.M., Hanson P., Weickert M.O., Franks S. (2019). Obesity and Polycystic Ovary Syndrome: Implications for Pathogenesis and Novel Management Strategies. Clin. Med. Insights Reprod. Health.

[10] Insenser M., Murri M., Del Campo R., Martínez-García M.Á., Fernández-Durán E., Escobar-Morreale H.F. (2018). Gut Microbiota and the Polycystic Ovary Syndrome: Influence of Sex, Sex Hormones, and Obesity. J. Clin. Endocrinol. Metab..

[11] Chu W., Han Q., Xu J., Wang J., Sun Y., Li W., Chen Z.-J., Du Y. (2020). Metagenomic Analysis Identified Microbiome Alterations and Pathological Association between Intestinal Microbiota and Polycystic Ovary Syndrome. Fertil. Steril..

[12] Zhou L., Ni Z., Cheng W., Yu J., Sun S., Zhai D., Yu C., Cai Z. (2020). Characteristic Gut Microbiota and Predicted Metabolic Functions in Women with PCOS. Endocr. Connect..

[13] Torres P.J., Ho B.S., Arroyo P., Sau L., Chen A., Kelley S.T., Thackray V.G. (2019). Exposure to a Healthy Gut Microbiome Protects against Reproductive and Metabolic Dysregulation in a PCOS Mouse Model. Endocrinology.

[14] Qi X., Yun C., Sun L., Xia J., Wu Q., Wang Y., Wang L., Zhang Y., Liang X., Wang L. (2019). Gut Microbiota-Bile Acid-Interleukin-22 Axis Orchestrates Polycystic Ovary Syndrome. Nat. Med..

[15] Durack J., Lynch S.V. (2019). The Gut Microbiome: Relationships with Disease and Opportunities for Therapy. J. Exp. Med..

[16] Tremellen K., Pearce K. (2012). Dysbiosis of Gut Microbiota (DOGMA)—A Novel Theory for the Development of Polycystic Ovarian Syndrome. Med. Hypotheses.

[17] Kelley S.T., Skarra D.V., Rivera A.J., Thackray V.G. (2016). The Gut Microbiome Is Altered in a Letrozole-Induced Mouse Model of Polycystic Ovary Syndrome. PLoS ONE.

[18] Akgül S., Düzçeker Y., Kanbur N., Derman O. (2018). Do Different Diagnostic Criteria Impact Polycystic Ovary Syndrome Diagnosis for Adolescents?. J. Pediatr. Adolesc. Gynecol..

[19] Adone A., Fulmali D.G. (2023). Polycystic Ovarian Syndrome in Adolescents. Cureus.

[20] Naz M.S.G., Tehrani F.R., Majd H.A., Ahmadi F., Ozgoli G., Fakari F.R., Ghasemi V. (2019). The Prevalence of Polycystic Ovary Syndrome in Adolescents: A Systematic Review and Meta-Analysis. Int. J. Reprod. Biomed..

[21] Aboeldalyl S., James C., Seyam E., Ibrahim E.M., Shawki H.E.-D., Amer S. (2021). The Role of Chronic Inflammation in Polycystic Ovarian Syndrome-A Systematic Review and Meta-Analysis. Int. J. Mol. Sci..

[22] Meczekalski B., Niwczyk O., Kostrzak A., Maciejewska-Jeske M., Bala G., Szeliga A. (2023). PCOS in Adolescents—Ongoing Riddles in Diagnosis and Treatment. J. Clin. Med..

[23] Parker J., O’Brien C., Hawrelak J., Gersh F.L. (2022). Polycystic Ovary Syndrome: An Evolutionary Adaptation to Lifestyle and the Environment. Int. J. Environ. Res. Public Health.

[24] Steegers-Theunissen R.P.M., Wiegel R.E., Jansen P.W., Laven J.S.E., Sinclair K.D. (2020). Polycystic Ovary Syndrome: A Brain Disorder Characterized by Eating Problems Originating during Puberty and Adolescence. Int. J. Mol. Sci..

[25] Garg D., Tal R. (2016). The Role of AMH in the Pathophysiology of Polycystic Ovarian Syndrome. Reprod. Biomed. Online.

[26] Ibáñez L., Díaz R., López-Bermejo A., Marcos M.V. (2009). Clinical Spectrum of Premature Pubarche: Links to Metabolic Syndrome and Ovarian Hyperandrogenism. Rev. Endocr. Metab. Disord..

[27] Risal S., Pei Y., Lu H., Manti M., Fornes R., Pui H.-P., Zhao Z., Massart J., Ohlsson C., Lindgren E. (2019). Prenatal Androgen Exposure and Transgenerational Susceptibility to Polycystic Ovary Syndrome. Nat. Med..

[28] Heidarzadehpilehrood R., Pirhoushiaran M., Abdollahzadeh R., Binti Osman M., Sakinah M., Nordin N., Abdul Hamid H. (2022). A Review on CYP11A1, CYP17A1, and CYP19A1 Polymorphism Studies: Candidate Susceptibility Genes for Polycystic Ovary Syndrome (PCOS) and Infertility. Genes.

[29] Spritzer P.M., Marchesan L.B., Santos B.R., Fighera T.M. (2022). Hirsutism, Normal Androgens and Diagnosis of PCOS. Diagnostics.

[30] Wang Y., Leung P., Li R., Wu Y., Huang H. (2022). Editorial: Polycystic Ovary Syndrome (PCOS): Mechanism and Management. Front. Endocrinol..

[31] Chang S., Dunaif A. (2021). Diagnosis of Polycystic Ovary Syndrome: Which Criteria to Use and When?. Endocrinol. Metab. Clin. N. Am..

[32] Polson D.W., Adams J., Wadsworth J., Franks S. (1988). Polycystic Ovaries—A Common Finding in Normal Women. Lancet.

[33] Fauser B.C.J.M., Tarlatzis B.C., Rebar R.W., Legro R.S., Balen A.H., Lobo R., Carmina E., Chang J., Yildiz B.O., Laven J.S.E. (2012). Consensus on Women’s Health Aspects of Polycystic Ovary Syndrome (PCOS): The Amsterdam ESHRE/ASRM-Sponsored 3rd PCOS Consensus Workshop Group. Fertil. Steril..

[34] Rosenfield R.L. (2020). Perspectives on the International Recommendations for the Diagnosis and Treatment of Polycystic Ovary Syndrome in Adolescence. J. Pediatr. Adolesc. Gynecol..

[35] Vassalou H., Sotiraki M., Michala L. (2019). PCOS Diagnosis in Adolescents: The Timeline of a Controversy in a Systematic Review. J. Pediatr. Endocrinol. Metab..

[36] Peña A.S., Metz M. (2018). What Is Adolescent Polycystic Ovary Syndrome?. J. Paediatr. Child. Health.

[37] Fulghesu A.M., Canu E., Casula L., Melis F., Gambineri A. (2021). Polycystic Ovarian Morphology in Normocyclic Non-Hyperandrogenic Adolescents. J. Pediatr. Adolesc. Gynecol..

[38] Peña A.S., Witchel S.F., Hoeger K.M., Oberfield S.E., Vogiatzi M.G., Misso M., Garad R., Dabadghao P., Teede H. (2020). Adolescent Polycystic Ovary Syndrome According to the International Evidence-Based Guideline. BMC Med..

[39] Witchel S.F., Oberfield S., Rosenfield R.L., Codner E., Bonny A., Ibáñez L., Pena A., Horikawa R., Gomez-Lobo V., Joel D. (2015). The Diagnosis of Polycystic Ovary Syndrome during Adolescence. Horm. Res. Paediatr..

[40] Ibáñez L., Oberfield S.E., Witchel S., Auchus R.J., Chang R.J., Codner E., Dabadghao P., Darendeliler F., Elbarbary N.S., Gambineri A. (2017). An International Consortium Update: Pathophysiology, Diagnosis, and Treatment of Polycystic Ovarian Syndrome in Adolescence. Horm. Res. Paediatr..

[41] Witchel S.F., Teede H.J., Peña A.S. (2020). Curtailing PCOS. Pediatr. Res..

[42] Cooney L.G., Dokras A. (2018). Beyond Fertility: Polycystic Ovary Syndrome and Long-Term Health. Fertil. Steril..

[43] Trent M., Gordon C.M. (2020). Diagnosis and Management of Polycystic Ovary Syndrome in Adolescents. Pediatrics.

[44] Fitzgerald S., DiVasta A., Gooding H. (2018). An Update on PCOS in Adolescents. Curr. Opin. Pediatr..

[45] Pkhaladze L., Barbakadze L., Kvashilava N. (2016). Myo-Inositol in the Treatment of Teenagers Affected by PCOS. Int. J. Endocrinol..

[46] D Genazzani A. (2014). Effects of a Combination of Alpha Lipoic Acid and Myo-Inositol on Insulin Dynamics in Overweight/Obese Patients with PCOS. Endocrinol. Metab. Syndr..

[47] Stein I.F., Leventhal M.L. (1935). Amenorrhea Associated with Bilateral Polycystic Ovaries. Am. J. Obstet. Gynecol..

[48] Dapas M., Dunaif A. (2022). Deconstructing a Syndrome: Genomic Insights Into PCOS Causal Mechanisms and Classification. Endocr. Rev..

[49] Rebar R., Judd H.L., Yen S.S., Rakoff J., Vandenberg G., Naftolin F. (1976). Characterization of the Inappropriate Gonadotropin Secretion in Polycystic Ovary Syndrome. J. Clin. Investig..

[50] Taylor A.E., McCourt B., Martin K.A., Anderson E.J., Adams J.M., Schoenfeld D., Hall J.E. (1997). Determinants of Abnormal Gonadotropin Secretion in Clinically Defined Women with Polycystic Ovary Syndrome1. J. Clin. Endocrinol. Metab..

[51] Dunaif A. (2016). Perspectives in Polycystic Ovary Syndrome: From Hair to Eternity. J. Clin. Endocrinol. Metab..

[52] Azziz R., Carmina E., Dewailly D., Diamanti-Kandarakis E., Escobar-Morreale H.F., Futterweit W., Janssen O.E., Legro R.S., Norman R.J., Taylor A.E. (2009). The Androgen Excess and PCOS Society Criteria for the Polycystic Ovary Syndrome: The Complete Task Force Report. Fertil. Steril..

[53] Dewailly D., Barbotin A.-L., Dumont A., Catteau-Jonard S., Robin G. (2020). Role of Anti-Müllerian Hormone in the Pathogenesis of Polycystic Ovary Syndrome. Front. Endocrinol..

[54] Dumont A., Robin G., Catteau-Jonard S., Dewailly D. (2015). Role of Anti-Müllerian Hormone in Pathophysiology, Diagnosis and Treatment of Polycystic Ovary Syndrome: A Review. Reprod. Biol. Endocrinol..

[55] Gilling-Smith C., Willis D.S., Beard R.W., Franks S. (1994). Hypersecretion of Androstenedione by Isolated Thecal Cells from Polycystic Ovaries. J. Clin. Endocrinol. Metab..

[56] Glueck C.J., Goldenberg N. (2019). Characteristics of Obesity in Polycystic Ovary Syndrome: Etiology, Treatment, and Genetics. Metabolism.

[57] Alvarez-Blasco F., Botella-Carretero J.I., Millán J.L.S., Escobar-Morreale H.F. (2007). Prevalence and Characteristics of the Polycystic Ovary Syndrome in Overweight and Obese Women. Obstet. Gynecol..

[58] Flannery C.A., Rackow B., Cong X., Duran E., Selen D.J., Burgert T.S. (2013). Polycystic Ovary Syndrome in Adolescence: Impaired Glucose Tolerance Occurs across the Spectrum of BMI: IGT in Adolescent Polycystic Ovary Syndrome. Pediatr. Diabetes.

[59] Hickey M., Doherty D.A., Atkinson H., Sloboda D.M., Franks S., Norman R.J., Hart R. (2011). Clinical, Ultrasound and Biochemical Features of Polycystic Ovary Syndrome in Adolescents: Implications for Diagnosis. Hum. Reprod..

[60] Ezeh U., Yildiz B.O., Azziz R. (2013). Referral Bias in Defining the Phenotype and Prevalence of Obesity in Polycystic Ovary Syndrome. J. Clin. Endocrinol. Metab..

[61] Ohashi K., Shibata R., Murohara T., Ouchi N. (2014). Role of Anti-Inflammatory Adipokines in Obesity-Related Diseases. Trends Endocrinol. Metab..

[62] Unamuno X., Gómez-Ambrosi J., Rodríguez A., Becerril S., Frühbeck G., Catalán V. (2018). Adipokine Dysregulation and Adipose Tissue Inflammation in Human Obesity. Eur. J. Clin. Investig..

[63] Su X., Peng D. (2020). Adipokines as Novel Biomarkers of Cardio-Metabolic Disorders. Clin. Chim. Acta.

[64] Nigro E., Scudiero O., Monaco M.L., Palmieri A., Mazzarella G., Costagliola C., Bianco A., Daniele A. (2014). New Insight into Adiponectin Role in Obesity and Obesity-Related Diseases. BioMed Res. Int..

[65] Wang Z.V., Scherer P.E. (2016). Adiponectin, the Past Two Decades. J. Mol. Cell Biol..

[66] Lainez N.M., Coss D. (2019). Obesity, Neuroinflammation, and Reproductive Function. Endocrinology.

[67] Engin A. (2017). The Pathogenesis of Obesity-Associated Adipose Tissue Inflammation. Adv. Exp. Med. Biol..

[68] Mannerås-Holm L., Leonhardt H., Kullberg J., Jennische E., Odén A., Holm G., Hellström M., Lönn L., Olivecrona G., Stener-Victorin E. (2011). Adipose Tissue Has Aberrant Morphology and Function in PCOS: Enlarged Adipocytes and Low Serum Adiponectin, but Not Circulating Sex Steroids, Are Strongly Associated with Insulin Resistance. J. Clin. Endocrinol. Metab..

[69] Diamanti-Kandarakis E., Dunaif A. (2012). Insulin Resistance and the Polycystic Ovary Syndrome Revisited: An Update on Mechanisms and Implications. Endocr. Rev..

[70] Kojta I., Chacińska M., Błachnio-Zabielska A. (2020). Obesity, Bioactive Lipids, and Adipose Tissue Inflammation in Insulin Resistance. Nutrients.

[71] Calcaterra V., Regalbuto C., Porri D., Pelizzo G., Mazzon E., Vinci F., Zuccotti G., Fabiano V., Cena H. (2020). Inflammation in Obesity-Related Complications in Children: The Protective Effect of Diet and Its Potential Role as a Therapeutic Agent. Biomolecules.

[72] Dumesic D.A., Oberfield S.E., Stener-Victorin E., Marshall J.C., Laven J.S., Legro R.S. (2015). Scientific Statement on the Diagnostic Criteria, Epidemiology, Pathophysiology, and Molecular Genetics of Polycystic Ovary Syndrome. Endocr. Rev..

[73] Dewailly D., Catteau-Jonard S., Reyss A.-C., Leroy M., Pigny P. (2006). Oligoanovulation with Polycystic Ovaries but Not Overt Hyperandrogenism. J. Clin. Endocrinol. Metab..

[74] Zeng X., Xie Y., Liu Y., Long S., Mo Z. (2020). Polycystic Ovarian Syndrome: Correlation between Hyperandrogenism, Insulin Resistance and Obesity. Clin. Chim. Acta.

[75] Goodarzi M.O., Carmina E., Azziz R. (2015). DHEA, DHEAS and PCOS. J. Steroid Biochem. Mol. Biol..

[76] Sender R., Fuchs S., Milo R. (2016). Are We Really Vastly Outnumbered? Revisiting the Ratio of Bacterial to Host Cells in Humans. Cell.

[77] Parker J., O’Brien C., Hawrelak J. (2022). A Narrative Review of the Role of Gastrointestinal Dysbiosis in the Pathogenesis of Polycystic Ovary Syndrome. Obstet. Gynecol. Sci..

[78] Yatsunenko T., Rey F.E., Manary M.J., Trehan I., Dominguez-Bello M.G., Contreras M., Magris M., Hidalgo G., Baldassano R.N., Anokhin A.P. (2012). Human Gut Microbiome Viewed across Age and Geography. Nature.

[79] Human Microbiome Project Consortium (2012). Structure, Function and Diversity of the Healthy Human Microbiome. Nature.

[80] Tremaroli V., Bäckhed F. (2012). Functional Interactions between the Gut Microbiota and Host Metabolism. Nature.

[81] Nicholson J.K., Holmes E., Kinross J., Burcelin R., Gibson G., Jia W., Pettersson S. (2012). Host-Gut Microbiota Metabolic Interactions. Science.

[82] Heianza Y., Ma W., Manson J.E., Rexrode K.M., Qi L. (2017). Gut Microbiota Metabolites and Risk of Major Adverse Cardiovascular Disease Events and Death: A Systematic Review and Meta-Analysis of Prospective Studies. J. Am. Heart Assoc..

[83] David L.A., Maurice C.F., Carmody R.N., Gootenberg D.B., Button J.E., Wolfe B.E., Ling A.V., Devlin A.S., Varma Y., Fischbach M.A. (2014). Diet Rapidly and Reproducibly Alters the Human Gut Microbiome. Nature.

[84] Mammadova G., Ozkul C., Yilmaz Isikhan S., Acikgoz A., Yildiz B.O. (2021). Characterization of Gut Microbiota in Polycystic Ovary Syndrome: Findings from a Lean Population. Eur. J. Clin. Investig..

[85] Lindheim L., Bashir M., Münzker J., Trummer C., Zachhuber V., Leber B., Horvath A., Pieber T.R., Gorkiewicz G., Stadlbauer V. (2017). Alterations in Gut Microbiome Composition and Barrier Function Are Associated with Reproductive and Metabolic Defects in Women with Polycystic Ovary Syndrome (PCOS): A Pilot Study. PLoS ONE.

[86] Jobira B., Frank D.N., Pyle L., Silveira L.J., Kelsey M.M., Garcia-Reyes Y., Robertson C.E., Ir D., Nadeau K.J., Cree-Green M. (2020). Obese Adolescents with PCOS Have Altered Biodiversity and Relative Abundance in Gastrointestinal Microbiota. J. Clin. Endocrinol. Metab..

[87] Li N., Li Y., Qian C., Liu Q., Cao W., Ma M., He R., Chen R., Geng R., Liu Y. (2020). Dysbiosis of the Saliva Microbiome in Patients with Polycystic Ovary Syndrome. Front. Cell Infect. Microbiol..

[88] Zhang J., Sun Z., Jiang S., Bai X., Ma C., Peng Q., Chen K., Chang H., Fang T., Zhang H. (2019). Probiotic Bifidobacterium Lactis V9 Regulates the Secretion of Sex Hormones in Polycystic Ovary Syndrome Patients through the Gut-Brain Axis. mSystems.

[89] Liu R., Zhang C., Shi Y., Zhang F., Li L., Wang X., Ling Y., Fu H., Dong W., Shen J. (2017). Dysbiosis of Gut Microbiota Associated with Clinical Parameters in Polycystic Ovary Syndrome. Front. Microbiol..

[90] Zeng B., Lai Z., Sun L., Zhang Z., Yang J., Li Z., Lin J., Zhang Z. (2019). Structural and Functional Profiles of the Gut Microbial Community in Polycystic Ovary Syndrome with Insulin Resistance (IR-PCOS): A Pilot Study. Res. Microbiol..

[91] Rizk M.G., Thackray V.G. (2021). Intersection of Polycystic Ovary Syndrome and the Gut Microbiome. J. Endocr. Soc..

[92] Zhao X., Jiang Y., Xi H., Chen L., Feng X. (2020). Exploration of the Relationship Between Gut Microbiota and Polycystic Ovary Syndrome (PCOS): A Review. Geburtshilfe Frauenheilkd.

[93] Zhu S., Zhang B., Jiang X., Li Z., Zhao S., Cui L., Chen Z.-J. (2019). Metabolic Disturbances in Non-Obese Women with Polycystic Ovary Syndrome: A Systematic Review and Meta-Analysis. Fertil. Steril..

[94] Wolf W.M., Wattick R.A., Kinkade O.N., Olfert M.D. (2018). Geographical Prevalence of Polycystic Ovary Syndrome as Determined by Region and Race/Ethnicity. Int. J. Environ. Res. Public Health.

[95] Vom Steeg L.G., Klein S.L. (2017). Sex Steroids Mediate Bidirectional Interactions Between Hosts and Microbes. Horm. Behav..

[96] Yurkovetskiy L., Burrows M., Khan A.A., Graham L., Volchkov P., Becker L., Antonopoulos D., Umesaki Y., Chervonsky A.V. (2013). Gender Bias in Autoimmunity Is Influenced by Microbiota. Immunity.

[97] Han Q., Wang J., Li W., Chen Z.-J., Du Y. (2021). Androgen-Induced Gut Dysbiosis Disrupts Glucolipid Metabolism and Endocrinal Functions in Polycystic Ovary Syndrome. Microbiome.

[98] Alesi S., Ee C., Moran L.J., Rao V., Mousa A. (2022). Nutritional Supplements and Complementary Therapies in Polycystic Ovary Syndrome. Adv. Nutr..

[99] Singh S., Pal N., Shubham S., Sarma D.K., Verma V., Marotta F., Kumar M. (2023). Polycystic Ovary Syndrome: Etiology, Current Management, and Future Therapeutics. J. Clin. Med..

[100] Thackray V.G. (2019). Sex, Microbes, and Polycystic Ovary Syndrome. Trends Endocrinol. Metab..

[101] Tabrizi R., Ostadmohammadi V., Akbari M., Lankarani K.B., Vakili S., Peymani P., Karamali M., Kolahdooz F., Asemi Z. (2022). The Effects of Probiotic Supplementation on Clinical Symptom, Weight Loss, Glycemic Control, Lipid and Hormonal Profiles, Biomarkers of Inflammation, and Oxidative Stress in Women with Polycystic Ovary Syndrome: A Systematic Review and Meta-Analysis of Randomized Controlled Trials. Probiotics Antimicrob. Proteins.

[102] Bhalla P., Rengaswamy R., Karunagaran D., Suraishkumar G.K., Sahoo S. (2022). Metabolic Modeling of Host–Microbe Interactions for Therapeutics in Colorectal Cancer. NPJ Syst. Biol. Appl..

[103] Shamasbi S.G., Ghanbari-Homayi S., Mirghafourvand M. (2020). The Effect of Probiotics, Prebiotics, and Synbiotics on Hormonal and Inflammatory Indices in Women with Polycystic Ovary Syndrome: A Systematic Review and Meta-Analysis. Eur. J. Nutr..

[104] Kaur I., Suri V., Sachdeva N., Rana S.V., Medhi B., Sahni N., Ahire J., Singh A. (2022). Efficacy of Multi-Strain Probiotic along with Dietary and Lifestyle Modifications on Polycystic Ovary Syndrome: A Randomised, Double-Blind Placebo-Controlled Study. Eur. J. Nutr..

[105] Chudzicka-Strugała I., Kubiak A., Banaszewska B., Zwozdziak B., Siakowska M., Pawelczyk L., Duleba A.J. (2021). Effects of Synbiotic Supplementation and Lifestyle Modifications on Women with Polycystic Ovary Syndrome. J. Clin. Endocrinol. Metab..

[106] Heshmati J., Farsi F., Yosaee S., Razavi M., Rezaeinejad M., Karimie E., Sepidarkish M. (2019). The Effects of Probiotics or Synbiotics Supplementation in Women with Polycystic Ovarian Syndrome: A Systematic Review and Meta-Analysis of Randomized Clinical Trials. Probiotics Antimicrob. Proteins.

[107] Giampaolino P., Foreste V., Di Filippo C., Gallo A., Mercorio A., Serafino P., Improda F.P., Verrazzo P., Zara G., Buonfantino C. (2021). Microbiome and PCOS: State-of-Art and Future Aspects. Int. J. Mol. Sci..

[108] Kwok K.O., Fries L.R., Silva-Zolezzi I., Thakkar S.K., Iroz A., Blanchard C. (2022). Effects of Probiotic Intervention on Markers of Inflammation and Health Outcomes in Women of Reproductive Age and Their Children. Front. Nutr..

[109] Ghanei N., Rezaei N., Amiri G.A., Zayeri F., Makki G., Nasseri E. (2018). The Probiotic Supplementation Reduced Inflammation in Polycystic Ovary Syndrome: A Randomized, Double-Blind, Placebo-Controlled Trial. J. Funct. Foods.

[110] Miao C., Guo Q., Fang X., Chen Y., Zhao Y., Zhang Q. (2021). Effects of Probiotic and Synbiotic Supplementation on Insulin Resistance in Women with Polycystic Ovary Syndrome: A Meta-Analysis. J. Int. Med. Res..

[111] Silvestris E., De Pergola G., Rosania R., Loverro G. (2018). Obesity as Disruptor of the Female Fertility. Reprod. Biol. Endocrinol..

